# Patients with diabetes struggling to afford food and control their HbA1c in food-insecure areas in Bronx, NY

**DOI:** 10.1017/S1368980024001666

**Published:** 2024-10-02

**Authors:** Earle C Chambers, Samantha R Levano, Nevin Cohen, Andrew R Maroko, Andrew Telzak, Cara Stephenson-Hunter, Kevin P Fiori

**Affiliations:** 1 Department of Family and Social Medicine, Albert Einstein College of Medicine, 1300 Morris Park Avenue, Bronx, NY 10461, USA; 2 CUNY Graduate School of Public Health & Health Policy, City University of New York, 55 W 125th St, New York, NY 10027, USA; 3 Department of Pediatrics, Albert Einstein College of Medicine, 3411 Wayne Avenue, Bronx, NY 10467, USA

**Keywords:** Food insecurity, Social needs, Diabetes mellitus, Primary health care, Community health, Disease management

## Abstract

**Objective:**

To characterise the association between risk of poor glycaemic control and self-reported and area-level food insecurity among adult patients with type 2 diabetes.

**Design:**

We performed a retrospective, observational analysis of cross-sectional data routinely collected within a health system. Logistic regressions estimated the association between glycaemic control and the dual effect of *self-reported and area-level* measures of food insecurity.

**Setting:**

The health system included a network of ambulatory primary and speciality care sites and hospitals in Bronx County, NY.

**Participants:**

Patients diagnosed with type 2 diabetes who completed a health-related social need (HRSN) assessment between April 2018 and December 2019.

**Results:**

5500 patients with type 2 diabetes were assessed for HRSN with 7·1 % reporting an unmet food need. Patients with self-reported food needs demonstrated higher odds of having poor glycaemic control compared with those without food needs (adjusted OR (aOR): 1·59, 95 % CI: 1·26, 2·00). However, there was no conclusive evidence that *area-level food insecurity alone was a significant predictor* of glycaemic control (aOR: 1·15, 95 % CI: 0·96, 1·39). Patients with self-reported food needs residing in food-secure (aOR: 1·83, 95 % CI: 1·22, 2·74) and food-insecure (aOR: 1·72, 95 % CI: 1·25, 2·37) areas showed higher odds of poor glycaemic control than those without self-reported food needs residing in food-secure areas.

**Conclusions:**

These findings highlight the importance of utilising patient- and area-level social needs data to identify individuals for targeted interventions with increased risk of adverse health outcomes.

## Graphical abstract

**Figure:**
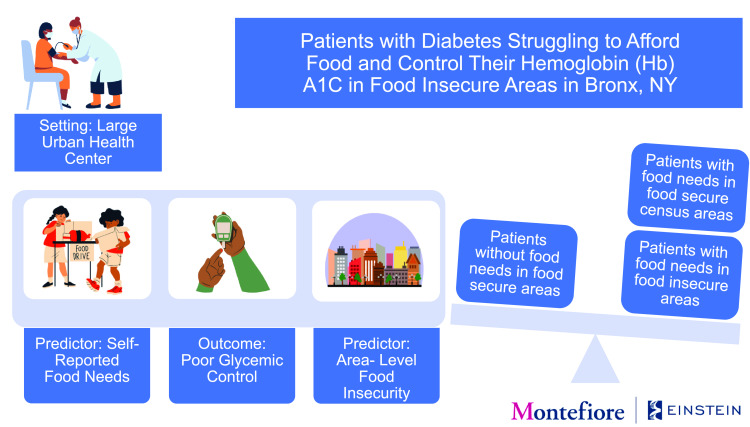

Previous research suggests that health behaviours and healthcare access required to support diabetes management can be undermined by health-related social needs (HRSN)^(1)^. HRSN are defined as the self-reported individual experiences of social risk factors at a specific moment in time^(2)^. These HRSN may include unstable and/or low-quality housing; unemployment; inability to pay for utilities, transportation or medication and food insecurity. Health systems have increasingly prioritised screening for HRSN in clinical settings^(3–6)^ following the release of the National Academy of Sciences, Engineering, and Medicine’s report titled, ‘Integrating Social Care into the Delivery of Health Care: Moving Upstream to Improve the Nation’s Health’ in 2019^(2)^. There remains no clear consensus, however, on how health systems should screen for HRSN, with some studies demonstrating a specified focus on screening populations with chronic conditions, including type 2 diabetes^(7)^.

Individuals with multiple HRSN have increased likelihood of poor diabetes control with each additional reported need^(8)^. Those with severe food insecurity alone are also known to be at greater risk for both developing diabetes and having poor glycaemic control^(9,10)^. Food insecurity is defined by Healthy People 2030 as a household-level socio-economic condition of limited or uncertain access to adequate food^(11)^. Results from a previous study within our health system showed that HRSN were associated with poor glycaemic control, with self-reported food needs among the strongest associations^(12)^. Unmet food needs are not only linked to worsened diabetes-related conditions but also to medication rationing, with patients forced to decide between purchasing food or purchasing medication^(13)^.

In the absence of self-reported data, studies often use area-level food insecurity as a proxy for individual-level food needs^(14)^. An NYC study of patients with diabetes found that residential proximity to healthy foods is associated with improved glycaemic control^(15)^. Prior studies have demonstrated the protective effects of close proximity to healthy neighbourhood resources (i.e. facilities, services and amenities that support the health and well-being of its residents) on factors related to glycaemic control such as healthy diets^(16,17)^, physical activity^(18)^, blood pressure^(19,20)^, BMI^(21)^, dyslipidaemia^(22)^ and blood sugar^(22)^. Longitudinal studies generally corroborate these inverse relationships^(16,23–27)^; however, results vary by subgroups (e.g. age, race, ethnicity, gender and socio-economic status)^(28–31)^. Some of this variability may result from individual barriers to adequate food that may work in concert with area level or neighbourhood food availability to increase the risk of adverse health outcomes.

Given the complex interactions between individual and neighbourhood factors, studies have yet to clearly identify what neighbourhood attributes are the most important predictors of diabetes and diabetes-related complications and for whom; however, most argue that healthier food options and access to amenities for physical activity promote optimal cardiovascular health^(32,33)^. Few studies have compared the relationship between individual-level food needs and area-level food insecurity in the geographic areas where patients reside. Most notable is a longitudinal study of patients with diabetes within a primary care network, which determined that individual-level food needs, but not residing in an area with access to healthy food options, was predictive of higher HbA1c; however, this study analysed individual and area-level measures of food security separately^(34)^.

In this study, we examine the dual relationship between self-reported food needs, defined by the patient during a routine clinical encounter and area-level food insecurity, defined by Feeding America, with glycaemic control among adult patients with type 2 diabetes in a large, urban health care system based in Bronx County, NY. We hypothesise that having a self-reported food need and living in a food-insecure area will result in the highest risk of poor glycaemic control.

## Methods

### Study design and sample

We performed a retrospective, cross-sectional analysis of HRSN data routinely collected within the health system between 1 April 2018 and 31 December 2019. The health system includes a network of hospitals as well as ambulatory primary and specialty care sites in Bronx and Westchester County, NY. The health system adapted a standardised ten-item HRSN assessment tool from a widely used, validated instrument, the Health Leads toolkit^(3)^, after an extensive pilot process involving key stakeholders^(35)^. The tool was self-administered, with nine versions available in the most preferred patient languages (e.g. English, Spanish, Albanian, Arabic, Armenian, French, Haitian, Portuguese, Bengali) and integrated into the health system’s electronic health record.

The HRSN assessment was first implemented at primary care sites in Bronx County, NY in April 2018. Each site determined the target population (e.g. new patients, pregnant patients, patients seen by particular clinicians) and frequency of assessment (e.g. during the initial visit, during the annual visit) based on staff availability, site-specific workflows and perceived burden of HRSN in the community. In addition to HRSN data, demographic information was also routinely collected as part of intake, including a patient’s primary residential address. If a patient was screened for HRSN multiple times during the study period, their most recent screen and primary residential address were reported. Residential addresses were extracted from the electronic health record and then geocoded to census tracts using geographic identifiers (GEOID).

Microsoft SQL Server, version 18, was used to extract data from the Epic EHR Data Warehouse. Patient addresses were geocoded using the New York State Street and Address Composite geocoding services tool for New York State addresses. Patient- and area-level measures were merged using the GEOID at the census tract.

### Measures

The primary outcome for this analysis was glycaemic control using HbA1c level (dichotomised as 0: HbA1c < 9·0, or good glycaemic control; 1: HbA1c ≥ 9·0, or poor glycaemic control) within the 3 months prior to the HRSN assessment. This HbA1c cut-point was selected to identify patients at the highest risk for poor glycaemic control and complications at the patient and area levels^(36)^. This HbA1c value is also consistent with the Healthy People 2030 goal to reduce the proportion of adults with diabetes with an HbA1c greater than 9 by the year 2030^(37)^. The primary predictor was a four-level categorical variable representing the dual effect of patient-level food needs and area-level food insecurity. The patient-level measure was defined as whether a patient self-reported a food need in the HRSN assessment tool (dichotomised as 0: ‘no’; 1: ‘yes,’) based on the answer to the following question: ‘In the last 12 months, did you worry that your food could run out before you got money to buy more?’ This measure was routinely collected as part of the assessment to best estimate household- or individual-level food needs (i.e. limited or uncertain access to adequate food due to lack of resources).

The area-level measure of food insecurity was sourced from Feeding America’s *Map the Meal Gap* study. Feeding America is the largest domestic hunger relief organisation in the USA with a network of 200 food banks serving approximately forty-six million people through food pantries, soup kitchens, shelters and other community-based agencies. Feeding America defines food insecurity as when people cannot access the food they need to live their fullest lives^(38)^. *Map the Meal Gap* generates local estimates of area-level food insecurity based on measures of income, poverty, unemployment, homeownership and prevalence of disability^(38)^. Feeding America sources the estimate of the total population from the American Community Survey (ACS) Five Year Estimates (2015–2019).

For the purposes of this study, we defined area-level food insecurity as the estimated percentage of the total population in food-insecure households in 2019 based on the census tracts (henceforth referred to as areas) where our patient population resided during the study period. We transformed the area-level food insecurity measure from a continuous to dichotomous predictor to facilitate clear interpretation and comparison against our individual-level predictor. In order to create an appropriate cut-point for this measure, we categorised the measure into sextiles based on the count of patients screened and identified the median rate of area-level food insecurity that coincided with an increase in median HbA1c within our sample of primarily Bronx County residents. Based on these findings, areas with a food insecurity prevalence below 15·5 % were categorised as food-secure and a prevalence at or above 15·5 % were considered food-insecure.

The primary four-level predictor variable was categorised as follows: 0: patients without self-reported food needs residing in food-secure areas; (1) patients without self-reported food needs residing in food-insecure areas; (2) patients with self-reported food needs residing in food-secure areas; (3) patients with self-reported food needs residing in food-insecure areas. Additional characteristics were collected for each patient assessed and included as covariates in analyses. These covariates reflect demographic and socio-economic confounders available in the electronic health record and utilised in our previous studies. Selected covariates included age (18–24; 25–44; 45–64; ≥ 65 years), sex (male, female), race and ethnicity (non-Hispanic white; Hispanic; non-Hispanic black; non-Hispanic Asian/Pacific Islander; non-Hispanic American Indian/Alaskan Native; missing indicator), preferred language (English; Spanish; Other; missing indicator) and health insurance at the time of assessment visit (Medicaid; Medicare; Commercial; Uninsured). Additionally, the analysis included the Elixhauser Comorbidity Index^(39)^ (0, 1, 2, 3 or more), which summarises a set of diagnostic criteria to assess and adjust for comorbidities (excluding diabetes); the index is assigned to the patient based on *International Classification of Diseases, Tenth Revision (ICD 10), Clinical Modification* codes assigned two years prior to the HRSN assessment.

### Mapping

**Fig. 1:**
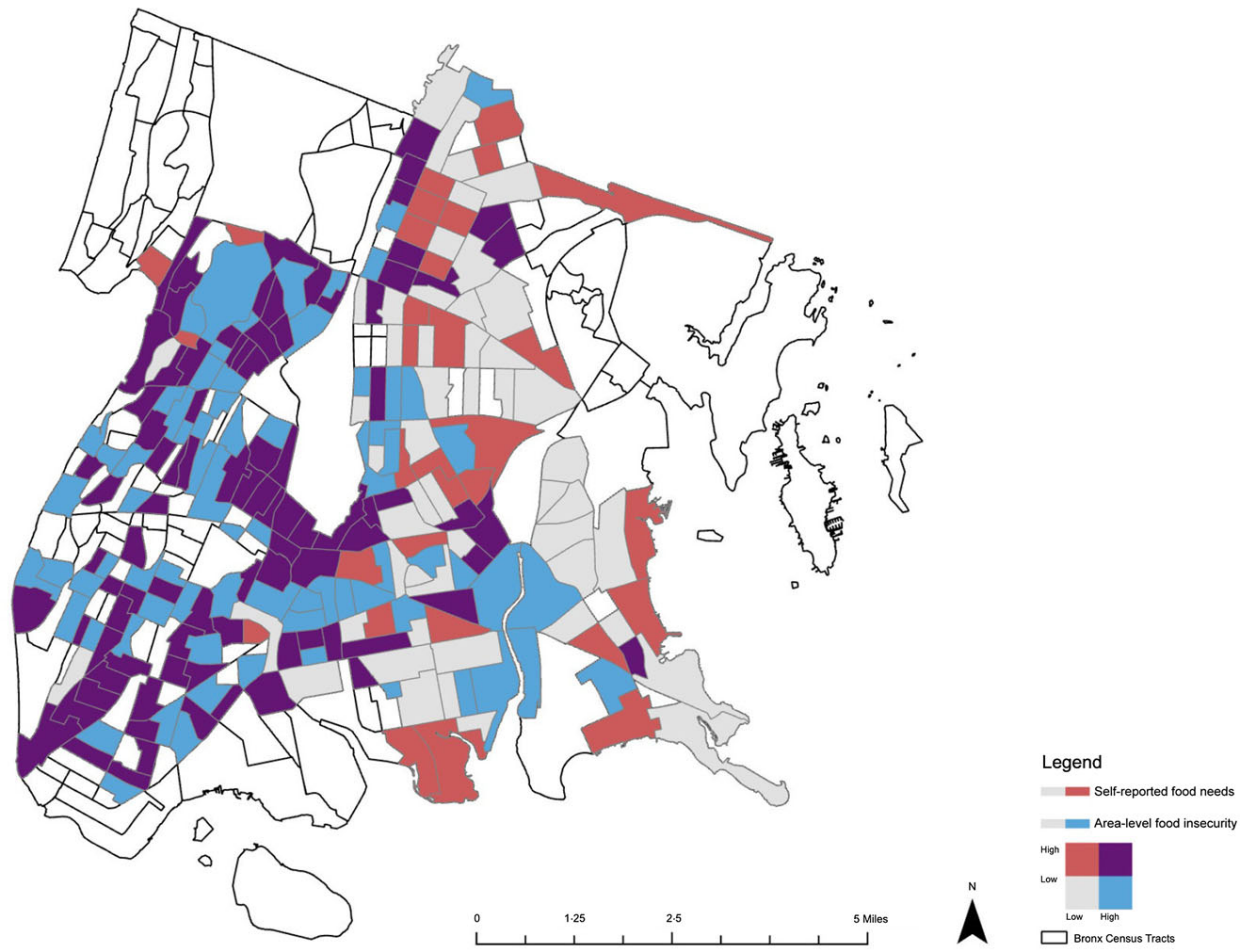
Prevalence of low and high self-reported food needs and area-level food insecurity, and confluence of both, per census tract in Bronx County, NY

We calculated the count of patients per census tract with self-reported food needs (1: ‘yes’) and divided this measure by the total count of patients assessed for HRSN per census tract to generate the prevalence for self-reported food needs in the study sample. The prevalence of self-reported food needs and area-level food insecurity per census tract was then used to generate a bivariate choropleth map in ArcGIS Pro (version 3.1, Esri Inc., Redlands, CA). Census tracts with five or fewer patient assessments were excluded from the map and represented in white. Self-reported prevalence was categorised into two discrete classes (low: less than or equal to the mean self-reported prevalence; high: greater than the mean self-reported prevalence) based on quantile distributions while area-level prevalence was categorised according to the pre-selected cut-point (low: less than or equal to 15·5 %; high: greater than or equal to 15·5 %). This resulted in a total of four unique colours displayed: grey (low prevalence of self-reported food needs and low prevalence of area-level food insecurity), blue (low prevalence of self-reported food needs and high prevalence of area-level food insecurity), red (high prevalence of self-reported food needs and low prevalence of area-level food insecurity) and purple (high prevalence of self-reported food needs and high prevalence of area-level food insecurity). The final bivariate choropleth map (Fig. 1) displays the highest and lowest values to visualise the spatial relationship between individual- and area-level measures.

### Statistical analyses

We used a two-step analytic approach with bivariate and multivariable logistic regression analyses to assess the relationship between glycaemic control and self-reported food needs and area-level food insecurity. First, we conducted analyses examining the association between (1) glycaemic control and self-reported food needs alone (Model 1) and (2) glycaemic control and area-level food insecurity alone (Model 2) to better understand the driving factor behind our primary predictor. We then conducted a bivariate logistic regression to estimate poor glycaemic control given the four-level primary predictor (Model 3). Next, multivariable logistic regressions were run for each model to determine the association between the predictor and poor glycaemic control while adjusting for covariates. Patients were excluded from the analysis if they were less than 18 years old, were missing GEOID, resided outside of Bronx County or did not meet previously defined inclusion criteria for predictor or outcome variables. All observations with missing values for the Elixhauser Comorbidity Index were excluded in the multivariable logistic regressions (*n* 4).

Statistical analyses were performed using STATA (version 15.1, StataCorp) in Q4 of 2022. All models accounted for the clustering of individuals by census tract by using the clustered sandwich estimator in STATA, which allows for intragroup correlation.

## Results

### Study and sample characteristics

Between April 2018 and December 2019, 54 854 unique patients completed an HRSN assessment at participating primary care sites within a Bronx, NY-based hospital system. Patients less than 18 years old (*n* 19 782), patients with missing GEOID (*n* 1941) and patients with GEOID outside of Bronx County, NY (*n* 6797) were excluded from the analysis. There were additional patients excluded without a type 2 diabetes diagnosis (ICD 10 code E11.X) in the two preceding years (*n* 45 724), without a response for self-reported food needs in the HRSN assessment tool (*n* 1344) and without a HbA1c test result in the 3 months prior to the completed assessment (*n* 36 165). In total, 49 354 patients were excluded from the screened population, with patients excluded due to one or more exclusion criteria. The final sample included 5500 unique patients.

Of these 5500 patients, half were above 65 years (50·0 %) followed by those aged 45–64 years (39·3 %) (Table 1). More than half of the patients (62·4 %) were female. Most patients self-identified as Hispanic (43·1 %) or non-Hispanic Black (29·9 %) with one in five (19·5 %) missing race or ethnicity data. Most of the population indicated that their preferred language was English (73·9 %), with an additional 22·9 % preferring Spanish. More than half were enrolled in Medicare (50·8 %) with the remaining patients enrolled in Medicaid (24·4 %) or Commercial Insurance (20·7 %) at the time of the assessment visit. According to the Elixhauser Comorbidity Index, 13·7 % of respondents had one diagnosed comorbidity, other than type 2 diabetes, 16·0 % had 2 and 68·1 % had ≥ 3 comorbidities.

**Table 1:** Descriptive characteristic of patients with good and poor glycaemic control (*n* 5500)

	All patients		Patients with good glycaemic control		Patients with poor glycaemic control	
	*n*	%	*n*	%	*n*	%
Total	5500	100·0	4317	78·5	1183	21·5
Age						
18–24	45	0·8	30	0·7	15	1·3
25–44	547	10·0	359	8·3	188	15·9
45–64	2159	39·3	1591	36·9	568	48·0
65+	2749	50·0	2337	54·1	412	34·8
Sex						
Male	2069	37·6	1542	35·7	527	44·6
Female	3431	62·4	2775	64·3	656	55·5
Race/ethnicity						
Non-Hispanic White	215	3·9	175	4·1	40	3·4
Hispanic	2369	43·1	1818	42·1	551	46·6
Non-Hispanic Black	1643	29·9	1313	30·4	330	27·9
Non-Hispanic American Indian/Alaskan Native	17	0·3	15	0·4	2	0·2
Non-Hispanic Asian/Pacific Islander	183	3·3	150	3·5	33	2·8
Missing	1073	19·5	846	19·6	227	19·2
Preferred language						
English	4065	73·9	3190	73·9	875	74·0
Spanish	1260	22·9	987	22·9	273	23·1
Other	154	2·8	123	2·9	31	2·6
Missing	21	0·4	17	0·4	4	0·3
Insurance						
Commercial	1140	20·7	860	19·9	280	23·7
Medicaid	1340	24·4	966	22·4	374	31·6
Medicare	2792	50·8	2336	54·1	456	38·6
Uninsured	228	4·2	155	3·6	73	6·2
Elixhauser Comorbidity Index						
0	118	2·2	86	2·0	32	2·7
1	762	13·7	554	12·8	208	17·6
2	872	16·0	682	15·9	190	16·1
3+	3744	68·1	2995	69·4	749	63·3
Missing	4	0·07	0	0·0	4	0·3
Self-reported food needs and area-level food insecurity						
Patients without food needs in food-secure areas	2180	39·6	1772	41·1	408	34·5
Patients without food needs in food-insecure areas	2929	53·3	2279	52·8	650	55·0
Patients with food needs in food-secure areas	119	2·2	80	1·9	39	3·3
Patients with food needs in food-insecure areas	272	5·0	186	4·3	86	7·3

Compared with 2020 US Census data for Bronx County, our study sample appeared older and more female; however, our health system routinely sees a higher distribution of women than men^(40)^. The study sample also reflects the high concentrations of Black or African American (44·3 %) and Hispanic or Latino (56·6 %) residents observed in Bronx County. Additionally, in our study sample, patients with poor glycaemic control appeared to be younger, more male (44·6 %) and insured through Medicaid (31·6 %) (Table 1).

Of those assessed for HRSN, 22·1 % reported one or more HRSN and 7·1 % reported an unmet food need. Most of the patients who completed the assessment did not report an unmet food need and resided in food-insecure areas (53·3 %), followed by food-secure areas (39·6 %). Meanwhile, more patients with unmet food needs resided in food-insecure areas (5·0 %) than food-secure areas (2·2 %) (Table 1).

### Mapping results

Figure 1 compares the prevalence of self-reported food needs with the prevalence of area-level food insecurity among census tracts in Bronx County, NY. Census tracts with five or fewer patient assessments, including those with no assessments, are represented in white. Census tracts with a high prevalence of self-reported food needs (> 7·1 %) and low area-level food insecurity rate (≤ 15·5 %) are represented in red. Meanwhile, census tracts with a low prevalence of self-reported food needs (≤ 7·1 %) and high area-level food insecurity rate (> 15·5 %) are represented in blue. Census tracts with both high prevalence of self-reported food needs (> 7·1 %) and high area-level food insecurity rate (> 15·5 %) are represented in purple. The census tracts with high prevalence of self-reported food needs and area-level food insecurity rates appear across Bronx County but are most concentrated in the southwest region. Meanwhile, census tracts with high prevalence of self-reported food needs and low area-level food insecurity appear more concentrated in eastern Bronx County.

### Bivariate analysis

Patients with self-reported food needs had an 80 % higher odds of poor glycaemic control compared with patients without self-reported food needs (OR: 1·80, 95 % CI: 1·44, 2·25) (Table 2). Patients residing in food-insecure areas also appeared to have a higher odds of poor glycaemic control compared with patients in food-secure areas; however, these findings were inconclusive (OR: 1·24, 95 % CI: 0·99, 1·53) (Table 2). Patients with self-reported food needs residing in food-secure areas had a 2·12 times higher odds of poor glycaemic control than patients without self-reported food needs residing in food-secure areas (OR: 2·12, 95 % CI: 1·45, 3·08) (Table 2). There was no conclusive evidence that patients without food needs residing in food-insecure areas had a significantly different risk of poor glycaemic control compared with patients without food needs in food-secure areas (OR: 1·24, 95 % CI: 1·00, 1·53). Patients with self-reported food needs residing in food-insecure areas, however, had a 2·01 times higher odds of poor glycaemic control than patients without food needs in food-secure areas (OR: 2·01, 95 % CI: 1·45, 2·79).

**Table 2:** Three bivariate logistic regressions of poor glycaemic control with self-reported food needs and area-level food insecurity (*n* 5500)

	OR	95 % Wald CI
Model 1: Self-reported food needs		
Predictor		
Patients without food needs	1·00	
Patients with food needs	1·80	1·44, 2·25*
Model 2: Area-level food insecurity		
Predictor		
Patients in food-secure census tract	1·00	
Patients in food-insecure census tract	1·24	0·99, 1·53
Model 3: Self-reported food needs and area-level food insecurity		
Predictor		
Patients without food needs in food-secure areas	1·00	
Patients without food needs in food-insecure areas	1·24	1·00, 1·53
Patients with food needs in food-secure areas	2·12	1·45, 3·08*
Patients with food needs in food-insecure areas	2·01	1·45, 2·79*

* Indicates a significant association at the *P* < 0·05 level between the predictor and outcome.

### Multivariable analysis

Self-reported food needs were a significant predictor with a 59 % higher odds of poor glycaemic control (adjusted OR (aOR): 1·59, 95 % CI: 1·26, 2·00) while adjusting for covariates; however, there remained no statistically significant association between area-level food insecurity and glycaemic control (aOR: 1·15, 95 % CI: 0·96, 1·39) (Table 3). After adjusting for covariates, patients with food needs residing in food-secure areas (aOR: 1·83, 95 % CI: 1·22, 2·74) and patients with food needs in food-insecure areas (aOR: 1·72, 95 % CI: 1·25, 2·37) showed an 83 % and 72 % higher odds of poor glycaemic control, respectively, compared with patients without self-reported food needs residing in food-secure areas (Table 3). Although there appeared to be a positive association with glycaemic control, there was no statistically significant difference in risk for those without food needs residing in food-insecure areas (aOR: 1·17, 95 % CI: 0·97, 1·40). In Model 3, patients who were uninsured had a higher odds of poor glycaemic control than patients with commercial insurance in the adjusted analysis (aOR: 1·48, 95 % CI: 1·12, 1·95). No additional covariates demonstrated a statistically significant difference in the odds of glycaemic control.

**Table 3:** Three multivariable logistic regressions of poor glycaemic control with self-reported food needs and area-level food insecurity (*n* 5496)

	OR	95 % Wald CI
Model 1: self-reported food needs^a^		
Predictor		
Patients without food needs	1·00	
Patients with food needs	1·59	1·26, 2·00*

Model 2: area-level food insecurity^b^		
Predictor		
Patients in food-secure census tract	1·00	
Patients in food-insecure census tract	1·15	0·96, 1·39

Model 3: self-reported food needs and area-level food insecurity		
Predictor		
Patients without food needs in food-secure areas	1·00	
Patients without food needs in food-insecure areas	1·17	0·97, 1·40
Patients with food needs in food-secure areas	1·83	1·22, 2·74*
Patients with food needs in food-insecure areas	1·72	1·25, 2·37*
Age		
18–24	1·00	
25–44	1·02	0·55, 1·89
45–64	0·72	0·39, 1·32
65+	0·41	0·22, 0·77
Sex		
Male	1·00	
Female	0·72	0·63, 0·83
Race/ethnicity		
Non-Hispanic White	1·00	
Hispanic	1·20	0·82, 1·74
Non-Hispanic Black	1·08	0·75, 1·55
Non-Hispanic American Indian/Alaskan Native	0·62	0·14, 2·78
Non-Hispanic Asian/Pacific Islander	0·83	0·45, 1·52
Missing	1·05	0·71, 1·56
Preferred language		
English	1·00	
Spanish	1·05	0·88, 1·26
Other	1·11	0·72, 1·70
Missing	1·29	0·43, 3·90
Insurance		
Commercial	1·00	
Medicaid	1·06	0·88, 1·28
Medicare	0·91	0·74, 1·11
Uninsured	1·48	1·12, 1·95*
Elixhauser Comorbidity Index		
0	1·00	
1	1·13	0·75, 1·69
2	0·93	0·62, 1·41
3+	0·98	0·66, 1·45

* Indicates a significant association at the *P* < 0·05 level between the predictor and outcome.

^a^ Adjusted odds ratios not reported for covariates in Model 1.

^b^ Adjusted odds ratios not reported for covariates in Model 2.

## Discussion

This study shows the dual association between having a food need and residing in a food-insecure area. Previous reports have demonstrated a significant positive association between self-reported food needs and poor glycaemic control^(12)^, but this study describes the additional influence of area-level food insecurity on glycaemic control. Our results suggest that patients with self-reported food needs are at the highest risk for poor glycaemic control whether they reside in a food-insecure area or not. In addition, the results indicate that patients with food needs residing in food-secure areas have the greatest risk of poor glycaemic control. Meanwhile, the bivariate map of the study sample highlights that there is not a direct spatial relationship between self-reported food needs and area-level food insecurity. While our study underscores the predictive value of individual-level data on glycaemic control, it does not diminish the importance of area-level data in understanding and addressing food insecurity within our patient population. Both types of data offer unique perspectives that complement each other and contribute to a more comprehensive understanding of the complexity of food insecurity and its impacts on health outcomes.

In this study, we determined that self-reported food needs demonstrated similar odds of poor glycaemic control (aOR: 1·59, 95 % CI: 1·26, 2·00) compared with that reported in a previous study in our health system (aOR: 1·50, 95 % CI: 1·19, 1·89), after adjusting for sociodemographic covariates^(12)^. These findings were also similar to that reported by Seligman et al. (aOR: 1·48, 95 % CI 1·07, 2·04) in safety net clinics in San Francisco and Chicago^(9,10)^, as well as additional studies supporting our claim that self-reported food need may drive the association with poor glycaemic control^(34)^. Self-reported food needs have been found to have a direct effect on glycaemic control as well as an indirect effect through increased stress, poorer diabetes self-care and lower social support^(41)^.

As previously stated, there are no studies that assess the dual association of self-reported food need and residing in a geographic area identified as food insecure by community-level proxy measures. Berkowitz et al. analysed individual-level and area-level data separately and concluded that living in an area with low physical food access was not associated with a difference in glycaemic control^(34)^. Similar conclusions were made by Birati et al. who examined an area-level measure of food insecurity for pregnant patients with either type I or type II diabetes and determined that there was no association between area-level poverty or area-level food insecurity and glycaemic control^(42)^. Another study limited to pregnant patients with either type I or type II diabetes determined that women living in food-insecure areas were more likely to enter pregnancy with poor glycaemic control, but more likely to achieve a decrease in HbA1c and achieve similar status to those in food-secure areas over time^(43)^.

Overall, our findings align with Cottrell et al., who reported on the lack of concordance between patient- and area-level social-risk data, highlighting the potential for ecological fallacy when using area-level data to identify patient-level needs^(44)^. This heterogeneity of risk can be helpful in informing multilevel interventions where teams including clinicians, health systems, and community partners are tasked with addressing food insecurity among patients and residents. Further investigation into patient-level food needs and area-level food insecurity is warranted to inform strategies that address food needs among patients in areas where food insecurity is low. Although more research is needed, it is possible that residing in a food-secure area might inadvertently limit access to affordable food and awareness of food assistance programmes. This potential may stem from the reliance on area-level measures to determine food costs, as well as variations in the perceived need for food assistance and vendor acceptance of nutrition and food assistance programmes as payment^(14)^. Expanding the use of patient-level and area-level data has the potential to facilitate the integration of geospatial analyses into programme planning to inform resource allocation and serve the families in greatest need. For example, health systems can access publicly available Supplemental Nutrition Assistance Program (i.e. the largest food and nutrition assistance programme in the USA) enrolment data by community district^(45)^. To demonstrate the potential use of this data, we compared the geographic distribution of our study sample with Supplemental Nutrition Assistance Program enrolment, not eligibility, and determined that patients with self-reported food needs were not always in areas where Supplemental Nutrition Assistance Program enrolment was highest (not shown). It is important to note that some demographic groups, often clustered in communities, have low levels of Supplemental Nutrition Assistance Program participation despite eligibility and need for nutrition assistance, which underscores the importance of considering the nuanced dynamics of access to assistance programmes and its intersection with community characteristics^(46)^. Future research should focus on investigating the mechanisms underlying the interplay between individual and area-level factors of food security. Understanding these mechanisms is essential for developing targeted interventions aimed at mitigating the adverse effects of food insecurity on health outcomes.

This study has limitations that should be considered when interpreting the results. The study design was cross-sectional and therefore could not measure causation or the impact of HRSN on glycaemic control over time. The HRSN assessment was also not universally implemented across the health system, with the decision to use the tool based on staff capacity, patient perceptions, practice volume and perceived patient risk, all of which contribute to the potential selection bias within the study sample. Additionally, the assessment was developed to identify ten different HRSN, not only food insecurity, which may present limitations in comprehensively measuring individual food needs. This study includes individual HRSN data of patients within one health system only, which limits our ability to compare individual- and area-level measures across all of Bronx County by excluding patients seen at other health systems. While it reflects the best representation of our available data, we do not account for the total Bronx population or total health system population in the bivariate choropleth map, which is another limitation of this analysis. In the map, we depict the prevalence of self-reported HRSN per census tract to account for the total population screened for HRSN within our health system and assess differences in HRSN screening density across tracts. In addition, area-level measures were based on a pre-selected cut-point; therefore, it is possible that the risk of poor glycaemic control could also be high at a lower prevalence of food insecurity.

Our results highlight the importance of not solely relying on area-level food insecurity data as a proxy for food needs in healthcare settings but rather the utility of combining these data to provide a more comprehensive picture of our patient population and the communities they reside within. In a recent review of food insecurity interventions in health systems, researchers showed that none of the studies utilised area-level data in their protocols or analysis. Future research should prioritise the screening of individual- or household-level food needs while also considering the value of area-level food insecurity data to assess proximity, affordability and access within the communities that patients reside to fully understand the food insecurity status of the patient population.
